# Using a Foreign Language Changes Medical Judgments of Preventative Care

**DOI:** 10.3390/brainsci11101309

**Published:** 2021-10-01

**Authors:** Sayuri Hayakawa, Yue Pan, Viorica Marian

**Affiliations:** 1 Department of Communication Sciences and Disorders, Northwestern University, Evanston, IL 60208, USA; v-marian@northwestern.edu; 2 Samuel Curtis Johnson Graduate School of Management, Cornell University, Ithaca, NY 14850, USA; yp388@cornell.edu

**Keywords:** foreign language, medical judgment, preventative healthcare, bilingualism, scope insensitivity, risk perception

## Abstract

Every day, multilinguals around the world make important healthcare decisions while using a foreign language. The present study examined how the use of a native vs. non-native language shapes evaluations and decisions about preventative care. Bilinguals were randomly assigned to evaluate a series of medical scenarios in either their native or non-native language. Each scenario described potential adverse effects of a medical condition and a preventative treatment, as well as the population risk of disease- or treatment-related complications. Participants judged the perceived negativity and likelihood of experiencing adverse effects and indicated how willing they would be to accept the preventative treatment. We found that bilinguals using a foreign language perceived disease symptoms and treatment side effects to be less negative than those using their native tongue. Foreign language users were also more likely to account for the objective risks associated with medical conditions and treatments when making decisions about preventative care. We conclude that the use of a native vs. foreign language changes how people evaluate the consequences of accepting and declining preventative treatment, with potential implications for millions of providers and patients who routinely make medical choices in their non-native tongue.

## 1. Introduction

Nearly 30% of all physicians in the United States (U.S.) are immigrants, working alongside millions of foreign-born nurses, technicians, and aids [1]. Among U.S. residents, over 20% report speaking a language other than English at home, with the estimate growing closer to 50% in the bigger cities [2]. Combined with the millions of multilinguals around the world who live their lives in a language other than their native tongue, it becomes clear that important decisions, such as those about our physical health, are routinely made while using a foreign language. How language impacts health outcomes has therefore garnered significant interest, with topics spanning the efficacy of interpreters and communication strategies ([3,4,5], see also [6] for review) to linguistic discrimination and perceptions of foreign accented doctors and patients ([7,8,9], see also [10] for review). One question that has received relatively less attention, however, is how evaluating health-related information in a foreign language impacts the clinical decision-making process.

Increasing evidence suggests that the use of a foreign language can systematically alter bilinguals’ judgments and preferences in domains ranging from moral judgment [11,12,13,14,15,16,17] and financial decision-making [18,19,20] to environmental conservation [21,22] and consumer choice [23,24,25]. Here, we explore whether the influence of language extends to medical contexts by examining how people make decisions regarding preventative healthcare when using a native vs. non-native tongue.

According to decision theory [26], individuals form preferences based on the expected utility of the available options—that is, the combination of how positive or negative a given outcome is perceived to be (i.e., the subjective value or utility) and how likely that outcome is perceived to be (i.e., the expected probability). Within this framework, an individual deciding whether to accept preventative treatment should consider how severe the consequences would be if a particular illness is contracted, as well as the probability of contracting the illness if no preventative measures are taken. These estimates could then be weighed against the perceived severity and probability of potential treatment complications to ultimately determine whether to accept preventative care. Considering the costs and benefits of multiple options, however, does not necessarily guarantee that the wisest decision will be made. In addition to the fact that we rarely have perfect knowledge of the stakes and probabilities associated with different outcomes, both subjective value and expected risk can be biased by emotional reactions and cognitive heuristics. Emotions can also affect the degree to which we account for expected utility when making decisions, resulting in substantial variability in people’s preferences and behaviors. In the following sections, we provide an overview of how medical judgements and decisions can be shaped by emotion, as well as how emotions, and subsequently preferences, can be shaped by language.

### 1.1. Evaluating Outcomes: How Bad Would It Be If Things Went Wrong?

The role of emotion in medical decision-making is perhaps most apparent when considering how individuals assign value to the available options. When deciding whether to get vaccinated for an illness, there are many different factors related to one’s health, finances, social and professional lives, and more that could be logically taken into account to estimate the value of each course of action. Sometimes, however, emotions are used as a substitute for reasoned evaluations, resulting in the relative value of one option over another becoming based on which one elicits a more positive or less negative feeling [27,28] (i.e., an “affect heuristic” [29]).

When making decisions regarding preventative healthcare, emotions such as fear, anxiety, and anger have been shown to influence a wide range of behaviors, including genetic testing for disease susceptibility [30], dietary habits [31], safe-sex practices [32], and willingness to accept preventative treatments that may introduce adverse effects of their own [33]. Making decisions that involve tradeoffs between negative outcomes of a disease and its treatment can be especially difficult as individuals are often disproportionately sensitive to the latter (i.e., “side effect aversion” [34,35,36,37]). Indeed, when informed of the possibility of treatment side effects, individuals will often prefer to take no action and instead face more severe consequences associated with the illness (i.e., an “omission bias” [38,39]). Waters et al. [36] observed that willingness to accept a preventative treatment declined with the “mere presence” of any negative side effect, but did not vary with the number of potential complications or with the likelihood of contracting the disease. It has also been found that ensuring comprehension of the treatment risks and benefits (e.g., through graphical displays) can reduce, but not eliminate side effect aversion [35], indicating that suboptimal decisions cannot be fully explained by imperfect knowledge or understanding. Instead, these findings are consistent with the idea that potential outcomes are often evaluated based on affective reactions rather than reasoned considerations.

We propose that one factor that could attenuate the perceived severity of adverse effects is the use of a foreign language. Bilinguals often report feeling less emotional when using a non-native language [40,41]—a finding that has been corroborated with physiological [42,43] and neural [44,45,46] evidence of attenuated emotional reactivity when processing foreign language stimuli. Bilinguals in psychotherapy settings reportedly switch to a non-native language to discuss traumatic or distressing topics [47], and verbally induced fear conditioning is reduced when using a foreign language [48]. Cross-linguistic differences may stem in part from language-dependent retrieval of memories [49,50,51,52] and the types of contexts in which native and foreign languages are acquired and used (see [53,54] for reviews). Marian and Kaushanskaya [50] observed that bilinguals express more intense affect when describing autobiographical memories in the language in which the memory took place. Differences in how bilinguals respond to and evaluate situations when using a native vs. non-native language can subsequently lead to different preferences and behaviors. For instance, when faced with moral dilemmas, bilinguals using a foreign language often express greater willingness to take emotionally aversive actions such as sacrificing one person’s life to save a group relative to those using their native language. More closely related to the health domain, Geipel, Hadjichristidis, and Klesse [22] found that innovative, but potentially aversive products (e.g., recycled water, insect-based food) were perceived as less disgusting in a foreign language, resulting in greater intended as well as actual consumption.

The effect of language on moral judgment may be partly attributable to a reduction in the vividness of mental imagery [17], which could subsequently reduce the emotional impact of difficult decisions. It has also been argued that effects of language on moral judgments may stem from less automatic and intuitive processing, rather than dampened emotion per se (e.g., [55,56]). Replicating earlier findings, Geipel et al. [15] observed that using a foreign language increased utilitarian responses to moral dilemmas and attenuated the perceived aversiveness of the decisions. Importantly, however, they found that the effect of language on moral judgment was not mediated by ratings of emotional distress. Furthermore, the authors observed that using a foreign language increased the perceived acceptability of violating social or moral norms, indicating that the influence of language on moral judgment may primarily stem from reduced activation of internalized norms (e.g., against causing harm to another person, even for the greater good).

Integrating the blunted norm-activation and attenuated emotion accounts, a recent study by Caldwell-Harris and Ayçiçeği-Dinn [57] investigated how using a foreign language affected ratings of agreement with ethical vs. selfish actions while measuring participants’ skin conductance responses (SCRs). Similar to Geipel et al. [15], the authors observed that participants using a foreign language expressed greater agreement with selfish actions than those using their native tongue. This finding lends support to the hypothesis that using a foreign language may reduce the salience of social and moral norms, which could contribute to foreign language users’ greater willingness to harm one individual to save a group (e.g., [12,13,16]). Additionally, while selfish statements generally elicited a larger stress response (i.e., higher SCRs) than ethical statements, the difference in SCRs was reduced in a foreign language. Notably, using a foreign language also led to an overall increase in physiological arousal. The authors therefore suggest that the added cognitive load and stress of using a non-native language may “swamp” emotional signals (i.e., “somatic markers” [58]), which would normally guide moral judgments. In other words, rather than merely dampening emotional responses, the anxiety and effort associated with using a less proficient language may mask the intuition-based gut feelings that enable quick and automatic judgments.

Such a mechanism could help reconcile the often-observed foreign language increase in utilitarian behavior with research suggesting that stress and cognitive load tend to elicit *less* utilitarian behavior [59,60]. Youssef et al. [59] found that when participants underwent a stress induction procedure, they tended to make fewer utilitarian decisions in response to high-conflict moral dilemmas (such as to sacrifice one person to save five) relative to controls. Likewise, Greene et al. [60] observed that increasing cognitive load with a concurrent digit-search task selectively interfered with utilitarian judgments. In both cases, the authors interpret their findings within the framework of a dual-process theory [61] of moral decision-making positing that judgments are made through a combination of relatively automatic, affective, and intuitive processes (i.e., System I) and more resource-dependent, deliberative, and analytical processes (i.e., System II). Based on the idea that stress and cognitive load should increase reliance on automatic processes, the consequent reduction in utilitarian choices has been taken as evidence that the “default” gut response when faced with sacrificial dilemmas is to abstain from taking the utility maximizing action—that is, to refuse to sacrifice one person to save a group. While at first glance, such an explanation seems to be at odds with the finding that the use of a more difficult foreign language *increases* utilitarianism, the apparent inconsistency could be resolved by Caldwell-Harris and Ayçiçeği-Dinn’s [57] proposal. For instance, while using a foreign language may indeed increase reliance on automatic or emotionally driven processes, any discomfort experienced when contemplating an aversive action may be attributed to the use of the foreign language rather than the choice itself, thereby minimizing its effectiveness as a cue.

The dual-process framework has also been invoked to suggest that the moral foreign language effect may stem, not from a reduction in emotion or intuition, but rather an increase in deliberative (System II) processing (see Hayakawa et al. [62] for discussion). This “enhanced deliberation” account is predicated on the idea that increasing perceptual or cognitive disfluency may enhance deliberative processes by signaling the need for more careful consideration. For instance, Alter et al. [63] found that participants completing the Cognitive Reflection Test (CRT [64]) were less likely to produce an intuitive, but incorrect response to misleading word problems when the text was more difficult to read. To the extent that a foreign language is processed less automatically and fluently than one’s native tongue, it may be more likely to engage careful, deliberative modes of thinking. If so, the greater willingness to sacrifice one life to save a group may result from increased deliberation and cost-benefit analysis rather than a reduction in the aversiveness of the sacrificial action itself. Along similar lines, it has been proposed that the foreign language increase in utilitarianism may be the result of language switching rather than the use of a foreign language per se. Oganian et al. [65] observed that bilinguals made more utilitarian moral judgments not only after switching from the native language to the foreign language, but also after switching from a foreign language to a native language (c.f. [12]). Given that language switching has been shown to engage domain-general cognitive control processes [66,67], the cognitive control required to inhibit a previously active language while activating another may carry over to subsequent moral judgments, resulting in greater deliberation and utilitarian preferences.

In sum, while using a foreign language can influence moral judgments and decisions, the mechanisms underlying existing foreign language effects have yet to be conclusively established. The robust evidence indicating that language modulates affective responses, however, provides a compelling basis to generate predictions regarding how using a foreign language may influence judgments in the medical domain, including emotionally charged evaluations of severity and risk.

### 1.2. Estimating Risks: What Is the Likelihood That Things Will Go Wrong?

In addition to guiding evaluations of outcomes, emotions can influence medical judgment by inflating or otherwise distorting the perceived likelihood of the potential consequences. The subjective value and expected probability of outcomes are often assumed to be theoretically independent, and yet there is substantial evidence that the estimated likelihood of experiencing an event is often biased by how beneficial or harmful that event is perceived to be [68]. For instance, the expected probability of catching a disease or experiencing treatment complications is often positively correlated with the perceived severity of the aversive outcome (e.g., [69,70]). Indeed, though risk is operationalized in the present study as the likelihood of experiencing adverse effects, the word “risk” itself is often associated with both the likelihood and severity of negative outcomes [71] (further highlighting the close correspondence between the two constructs). The influence of “affect heuristics” [29] can be observed even when individuals are explicitly provided with information regarding the prevalence of medical conditions (i.e., “population risks” [72]).

To the extent that using a foreign language attenuates the perceived negativity of potential outcomes, it may subsequently reduce the perceived likelihood of aversive events relative to when judgments are made in a native language. Indeed, it has been found that individuals evaluating the risks and benefits of potential hazards (e.g., nuclear energy, nanotechnology) perceive less risk and greater benefit when using a foreign language compared to a native language [73]. Such differences in risk perception may help account for a number of findings suggesting that using a foreign language can increase willingness to engage in novel and/or risky behaviors (e.g., [18,19,20,22,74]).

The role of emotion in language-dependent risk taking has also been explored in a number of recent neuroimaging studies (e.g., [75,76]). In one functional Magnetic Resonance Imaging (fMRI) study, He et al. [75] presented unbalanced bilinguals with a series of gambling decisions. Each gamble was followed by positively- or negatively-valenced written feedback signaling a monetary gain (e.g., “Wonderful! +$10”) or loss (e.g., “Terrible! −$3”) in either the native language (Chinese) or foreign language (English). Consistent with prior behavioral evidence indicating that judgments of risky events may be more positively biased in a non-native tongue [73], the authors found that positive feedback elicited a greater tendency to gamble in the foreign language than in the native language, which was in turn associated with greater activation of the right hippocampus (implicated in both declarative memory [77,78] and emotion regulation [79,80]). Relative to neutral feedback, negative feedback elicited greater functional connectivity between the dorsolateral prefrontal cortex (dlPFC) and visual cortex in the foreign language, but not in the native language. Given the role of the dlPFC in cognitive control processes [81,82], as well as analytic reasoning [83], the authors propose that using a foreign language may engage increased control processes to divert attention away from negative stimuli.

### 1.3. Weighing Costs and Benefits: Will You Rely on Intuition or Data?

In addition to directly modulating perceptions of risk, affective responses can influence the degree to which individuals are sensitive to actual or perceived probabilities when forming judgments and preferences. Colomé et al. [69] found that decisions to recommend preventative treatments for highly negative medical conditions (e.g., cancer) were primarily guided by affective evaluations (severity, worry) rather than the likelihood of contracting the disease. Decisions regarding less severe and worrisome conditions (e.g., hypertension), on the other hand, were more impacted by estimates of risk. The greater tendency to neglect probabilistic information when making emotional decisions is consistent with the moderating effect of emotion on “scope insensitivity” [84,85,86,87]. As it pertains to health risks, scope insensitivity (or embedding) refers to how individuals are often insufficiently sensitive to the magnitude of risk reduction when determining the value of treatments, policies, consumer products, and so on [88]. For instance, an individual may be willing to pay $500 to reduce the risk of a disease from 20% to 10% (i.e., a 10% reduction), and yet may only be willing to spend $600 to reduce the risk from 30% to 10% (i.e., a 20% reduction). In other words, twice the risk reduction would not be valued at twice the price. Rottenstreich and Hsee [86] find that individuals are especially insensitive to magnitude when evaluating high-affect outcomes (such as a painful electric shock) relative to lower-affect outcomes (such as a monetary loss, i.e., an “affect gap”, see [87]). The authors suggest that, for negative events, any deviation from impossibility to some possibility inspires fear, causing individuals to inflate the likelihood of small probabilities. At the other end, deviation from certainty to some possibility inspires hope, resulting in underestimations of high probabilities. Stronger emotional responses may thus flatten the probability-weighting function, resulting in reduced sensitivity to relative degrees of risk. Chang and Pham [84] similarly observed a relationship between scope insensitivity and psychological distance, with greater psychological proximity to the situation (temporally, socially, or physically) resulting in greater insensitivity. To the extent that a foreign language induces a more psychologically or emotionally distant mindset, the prospect of aversive medical events may elicit less fear, making people more likely to account for relative magnitudes of outcome severity and risk. In other words, using a foreign language may increase sensitivity to expected utility when making difficult medical decisions.

### 1.4. The Present Study

The goal of the present study was to assess the impact of using a native vs. a foreign language on judgments of preventative medical care, with a particular emphasis on (1) subjective evaluations of aversive outcomes (i.e., perceived negativity), (2) expected probabilities of aversive outcomes (i.e., perceived risk), and (3) sensitivity to expected utility when determining willingness to accept preventative treatment. Bilingual participants were presented with a series of hypothetical scenarios describing adverse effects associated with a medical condition and a preventative treatment. To determine the extent to which participants account for known probabilities when estimating their personal likelihood of experiencing adverse outcomes (i.e., “personal risk”), they were additionally informed of the objective likelihood (or “population risk”) of experiencing either disease symptoms or treatment complications. We predicted that the prospect of undesirable outcomes would elicit a weaker emotional response in a foreign language relative to a native tongue, reducing the perceived severity and likelihood of adverse effects and increasing sensitivity to expected utility. To test these predictions, participants were randomly assigned to complete the entire experiment in either the native (Chinese) or foreign language (English).

## 2. Materials and Methods

### 2.1. Participants

One-hundred-sixty native Mandarin Chinese speakers participated in the experiment (54% female; average age = 25.77, *SD* = 3.91). Participants were recruited through social media and the survey was administrated online using the Qualtrics platform [89]. Each participant was compensated with a $10 Amazon gift card and screened for eligibility prior to data collection. All participants reported higher proficiency in Mandarin than in English and had acquired Mandarin earlier than English. To ensure adequate proficiency in Mandarin and English to complete the experiment, participation was restricted to native Mandarin speakers residing in the United States who were either enrolled in or had graduated from a U.S. university. Proficiency in each language was confirmed with the *Language Experience and Proficiency Questionnaire* (*LEAP-Q,* [90]) using eleven-point (0-10) scales with corresponding labels (none, very low, low, fair, slightly less than adequate, adequate, slightly more than adequate, good, very good, excellent, perfect).

Average proficiency (aggregated across speaking, understanding, and reading) was high in both Mandarin (*M* = 9.65, *SD* = 0.85) and English (*M* = 7.99, *SD* = 1.18) and was significantly higher in Mandarin than in English (*p* < 0.0001). Participants were randomly assigned to complete the study in either their native language (Mandarin; *n* = 76) or their foreign language (English; *n* = 84), and reported a score of 6 or higher in the assigned language. The two groups did not differ in their age, gender distribution, Mandarin age of acquisition or reading acquisition, English age of acquisition or reading acquisition, Mandarin speaking, understanding, or reading proficiency, or English understanding or reading proficiency. Self-reported English speaking proficiency, however, was higher in the native language condition (see Table 1 for participant demographics). English speaking proficiency was controlled in follow-up analyses to confirm the robustness of all reported effects.

### 2.2. Design

Participants were presented with ten medical scenarios describing adverse effects associated with a medical condition and a preventative treatment, along with population risks associated with either the disease or the treatment. Each scenario was randomly paired with one of ten probabilities in the range from 2% to 30% (“low population risk”) or from 70% to 98% (“high population risk”) at 7% intervals. The study therefore followed a mixed within- and between-subject design, with a within-subject variable of Population Risk, and between-subject variables of Risk Condition (i.e., known probabilities for the disease or treatment), and Language (native Mandarin vs. foreign English). Language and Risk Condition were fully crossed, with each participant randomly assigned to one of four possible conditions: (a) Mandarin + disease population risk; (b) Mandarin + treatment population risk; (c) English + disease population risk; and (d) English + treatment population risk. The present study was conducted as part of a larger project, which included a separate investigation on language-dependent beliefs reported in Hayakawa, Pan, and Marian [91].

### 2.3. Stimuli and Procedure

All stimuli were originally created in English and then translated into Mandarin by a native Mandarin–English bilingual. Another Mandarin–English bilingual then back-translated the stimuli to English and the two English versions were compared [92]. Disagreements in translation were resolved through discussions among the two translators, a third Mandarin–English bilingual, and the authors. The mean Flesch Reading Ease score [93] for the scenarios was 53.09 (*SD* = 7.29; range: 37.12–62.61), which corresponds to a 10th to 11th grade reading level on the Flesch–Kincade scale.

After providing informed consent, participants were randomly assigned to read and respond to 10 medical scenarios in either their native (Mandarin) or non-native (English) language. No time limit was imposed in order to allow participants to complete the study at their own pace. Each scenario described a medical condition and a preventative medical procedure, including potential adverse effects associated with both. The full text of scenarios and data are available at https://osf.io/xue6t/ (accessed on 21 September 2021).

#### 2.3.1. Structure of Scenarios

- One sentence describing a potential medical problem: *You find out that millions of people are likely to get sick from the flu this year.*
- Two sentences describing adverse effects associated with declining preventative treatment (i.e., disease symptoms): *If you get the flu, you may experience a number of unpleasant symptoms such as a sore throat and fever. It could even turn into pneumonia, which can cause severe body aches and difficulty breathing.*
- One sentence describing a benefit of accepting preventative treatment: *You will greatly reduce your chance of catching the flu if you get a flu shot…*
- Three sentences describing adverse effects associated with accepting preventative treatment (i.e., treatment complications): *… but there are risks involved in getting the injection. Specifically, there may be soreness at the injection site. You may also experience weakness in your arms, making it difficult to perform normal tasks. You may also have allergic reactions to the shot, and experience negative symptoms such as difficulty breathing.*
- Within each language condition, half of the participants were provided with the population risk associated with declining preventative care (i.e., disease symptoms). These participants read the following information after the description of the medical condition: *According to estimates, approximately x% of people will experience one or more of the aforementioned negative effects if they do not receive the flu shot as preventative care.*
- The remaining participants were provided with the population risk associated with accepting preventative care (i.e., treatment complications). These participants read the following information after the description of the preventative treatment: *According to estimates, approximately x% of people will experience one or more of the aforementioned negative effects if they receive the flu shot as preventative care.*
- Each of the 10 scenarios was randomly paired with a “low population risk” (2%, 9%, 16%, 23%, or 30%) or a “high population risk” (70%, 77%, 84%, 91%, or 98%). Assignment of risks to scenarios, as well as the order of scenarios was randomized for each participant.

#### 2.3.2. Measures

After each scenario, participants made the following judgments on 0–100 scales:

- Willingness to accept preventative treatment: *How willing would you be to get the flu shot as preventative care? (0 = Not willing at all; 100 = Extremely willing)*
- Perceived (1) negativity and (2) risk of experiencing adverse effects associated with declining preventative care: *As mentioned above, choosing NOT TO get the flu shot as preventative care can cause some negative effects (sore throat, fever, pneumonia, severe body aches, and death).*
  ▪ *How bad do you think these negative effects are? (0 = Not bad at all; 100 = Extremely bad)*
  ▪ *How likely do you think it is that you would experience these negative effects? (0 = Not likely at all; 100 = Extremely likely)*
- Perceived (1) negativity and (2) risk of experiencing adverse effects associated with accepting preventative care: *As mentioned above, choosing TO get the flu shot as preventative care can also cause some negative effects (soreness, weakness, difficulty performing tasks, allergic reaction, difficulty breathing).*
  ▪ *How bad do you think these negative effects are? (0 = Not bad at all; 100 = Extremely bad)*
  ▪ *How likely do you think it is that you would experience these negative effects? (0 = Not likely at all; 100 = Extremely likely)*

After responding to all 10 scenarios, participants were administered the *LEAP-Q* [90] to assess language background.

## 3. Predictions and Analyses

We began by examining predictors of perceived negativity and perceived risk, followed by willingness to accept preventive care. Three separate linear mixed-effects models were constructed for each outcome variable to test the following predictions:

*Perceived Negativity*

- **Hypothesis 1 (H1).** Using a foreign language will attenuate the perceived negativity of potential adverse effects (i.e., disease symptoms and treatment complications).

*Perceived Risk*

- **Hypothesis 2 (H2).** Using a foreign language will reduce the perceived risk of potential adverse effects.
- **Hypothesis 3 (H3).** Using a foreign language will increase sensitivity to known population risks—that is, the effect of *population risk* on perceived *personal* risk should be greater in the foreign language than in the native language, particularly when population risks are directly relevant to the judgement (e.g., perceived personal risk of treatment complications based on known population risk of treatment complications).

*Willingness to Accept Preventative Care*

- **Hypothesis 4 (H4).** Using a foreign language will increase sensitivity to the *relative expected utility* of accepting vs. declining preventative treatment—that is, the degree to which the expected harm of disease symptoms exceeds that of treatment complications.

For all models, we began with the maximal random effects structure [94], including random intercepts for participant and scenario and by-participant and by-scenario random slopes for all fixed effects, as justified by the design. If convergence errors were encountered, the maximally converging model was identified by first removing random correlations, and then sequentially removing random slopes accounting for the least amount of variance until convergence was achieved. Contrasts for fixed effects were effect-coded and weighted by the number of responses in each condition (e.g., Language was coded as Chinese: −0.52 vs. English: +0.48 to account for the fact that there were fewer participants in the Chinese condition (*n* = 76) than in the English condition (*n* = 84)). Family-wise error rates were controlled in follow-up tests with Holm–Bonferroni-adjusted *p*-values.

Models for perceived negativity and perceived risk included the following fixed effects (with all interactions):

- **Language**. Native (Chinese): −0.52 vs. Foreign (English): +0.48;
- **Medical Event** (i.e., the outcome being evaluated). Disease Symptoms: −0.50 vs. Treatment Complications: +0.50;
- **Relevance** (of population risk to judgment). Irrelevant (e.g., evaluating the disease with knowledge of population treatment risks): −0.50 vs. Relevant (e.g., evaluating the disease with knowledge of population disease risks): +0.50;
- **Population Risk** (continuous measure of population risk).

The maximally converging model for each outcome variable included a random intercept for participant and scenario, as well as a by-participant random slope for Medical Event. The model for perceived negativity additionally included by-scenario random slopes for Language, Medical Event, and Relevance, and the model for perceived risk included by-scenario random slopes for Medical Event and Relevance.

The model for willingness to accept preventative treatment included the following fixed effects (with all interactions):

- **Language**. Native (Chinese): −0.52 vs. Foreign (English): +0.48;
- **Risk Condition**. Knowledge of population risks for the disease: −0.47 vs. Treatment: +0.53;
- **Relative Treatment Utility** (continuous measure of expected harm of declining treatment—expected harm of accepting treatment).

The maximally converging model additionally included random intercepts for participant and scenario, as well as by-scenario random slopes for Language and Risk Condition. To calculate the expected harm of declining treatment, we multiplied the perceived negativity of disease symptoms by the perceived risk of experiencing those symptoms. For instance, if a set of disease symptoms received a negativity rating of 80 out of 100 and the perceived risk of experiencing those symptoms was 50%, the expected harm of that disease would be 40. Likewise, the expected harm of accepting treatment was calculated by multiplying the perceived negativity of treatment complications by the perceived risk of treatment complications. The expected harm of accepting treatment was then subtracted from that of declining treatment to obtain a measure of Relative Treatment Utility (with positive values indicating that the perceived benefit of accepting treatment outweighed the costs).

We report all main and interactive effects of Language (native, foreign). Independent effects of Medical Event (disease, treatment) can be found in Appendix A. See Table A2 and Table A3 in the Appendix B for full parameter estimates grouped by outcome variable.

## 4. Results

### Effects of Language on Perceived Negativity, Perceived Risk, and Willingness to Accept Preventative Treatment

**Finding** **1:** *Using a foreign language reduces the perceived negativity of adverse effects (H1).*

**Figure 1:**
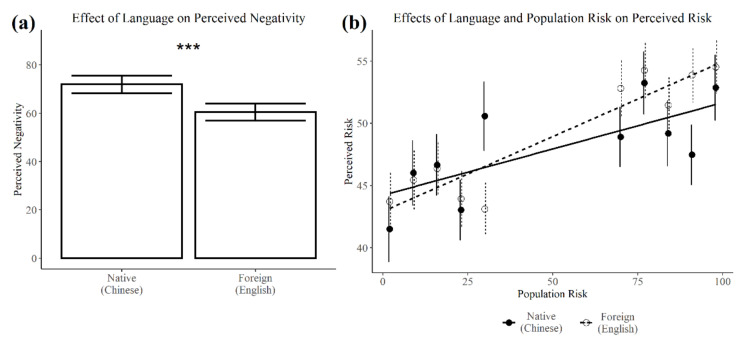
Effects of language on perceived negativity and perceived risk. Using a foreign language (**a**) attenuated the perceived negativity of aversive outcomes and (**b**) increased sensitivity to population risks when estimating personal risks. Error bars represent standard error. *** *p* < 0.001.

Aversive outcomes (disease symptoms, treatment complications) were perceived as more negative in the native language (*M* = 71.81, *SD* = 11.92) than in the foreign language (*M* = 60.51, *SD* = 12.31; see Figure 1a and Table 2). This main effect of Language was not moderated by Medical Event, Population Risk, or Relevance, suggesting that the aversiveness of potential outcomes was consistently attenuated when using a non-native tongue.

**Finding** **2:** *Using a foreign language increases sensitivity to known population risks when estimating personal risks (H3).*

Using a foreign language did not influence the overall level of perceived risk (H2), but instead increased sensitivity to known population risks (resulting in a significant interaction between Language and Population Risk, see Figure 1b and Table 2). In other words, the known prevalence of adverse outcomes within the population had a greater impact on bilinguals’ perceptions of personal risk when they were using a foreign language. Though we expected that using a foreign language would be especially likely to increase sensitivity to known risks when they were directly relevant to the judgment (e.g., perceived risk of treatment complications with knowledge of population treatment risks), the three-way interaction between Language, Population Risk, and Relevance was not significant (*p* = 0.252). Planned follow-up comparisons, however, revealed that using a foreign language increased sensitivity to relevant (*Estimate* = 0.08, *SE* = 0.04, *t*(1411.24) = 2.19, *p* = 0.028), but not irrelevant population risks (*Estimate* = 0.03, *SE* = 0.03, *t*(1410.28) = 0.79, *p* = 0.428).

Similar effects of language on perceived negativity and risk were found when the continuous measure of Population Risk was replaced with a categorical variable of low (2%, 9%, 16%, 23%, 30%) vs. high (70%, 77%, 84%, 91%, 98%) Population Risk. For perceived negativity, a significant main effect of Language revealed that using a foreign language attenuated the perceived severity of adverse outcomes (*Estimate* = −11.35, *SE* = 2.01, *t*(92.77) = −5.65, *p* < 0.001). For perceived risk, a significant Language × Population Risk interaction confirmed that using a foreign language increased sensitivity to population risks when estimating personal risks (*Estimate* = 4.65, *SE* = 1.75, *t*(2859.68) = 2.65, *p* = 0.008).

**Finding** **3:** *Using a foreign language increases sensitivity to expected utility when determining willingness to accept preventative treatment (H4).*

**Figure 2:**
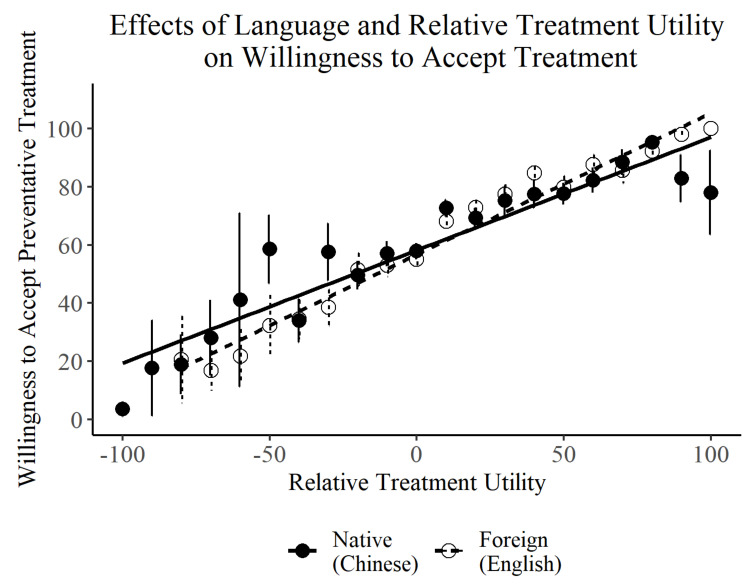
Effects of language and relative treatment utility on willingness to accept preventative treatment. Relative treatment utility was calculated as the expected harm of the disease (disease negativity × disease risk) minus the expected harm of the treatment (treatment negativity × treatment risk). Positive values indicate that the expected harm of the disease exceeded that of the treatment. Willingness to accept treatment increased with greater relative treatment utility, particularly when using a foreign language (dashed, white) compared to a native language (solid, black). Error bars represent standard errors within each 10-point bin from −100 (treatment harm far exceeds disease harm) to +100 (disease harm far exceeds treatment harm).

Overall, willingness to accept preventative treatment did not significantly differ between bilinguals using a native (*M* = 65.41, *SD* = 34.98) vs. foreign language (*M* = 64.99, *SD* = 32.07; *Estimate* = −1.93, *SE* = 2.96, *t*(38.24) = −0.65, *p* = 0.518). There was, however, a positive main effect of Relative Treatment Utility (*Estimate* = 0.39, *SE* = 0.02, *t*(1581.88) = 18.23, *p* < 0.001), as well as a significant interaction between Relative Treatment Utility and Language (*Estimate* = 0.10, *SE* = 0.04, *t*(1540.26) = 2.48, *p* = 0.013; see Figure 2). In other words, willingness to accept preventative care increased with the degree to which the expected harm of the disease exceeded that of the treatment, and this was especially the case when using a foreign language. The effects of Language and Relative Treatment Utility were not moderated by whether population risks were known for the treatment vs. the disease (*p* > 0.05 for all interactions with Risk Condition).

Though it was our prediction that using a foreign language would increase sensitivity to the overall relative utility of accepting the treatment, we followed up with two separate analyses to assess sensitivity to relative risk (disease risk—treatment risk) and relative negativity (disease negativity—treatment negativity). The main effects of both Relative Risk and Relative Negativity were highly significant (see Table 3), as were the simple effects for each language (*p* < 0.0001 for the effects of both Relative Risk and Relative Negativity for both the native and foreign language). While sensitivity to both variables was numerically greater in the foreign language, the moderating effect of language only reached significance for Relative Risk.

Including mean centered English speaking proficiency, Flesch Reading Ease scores, and all interactions as model covariates did not meaningfully alter the effects of language on perceived negativity, perceived risk, or willingness to accept preventative treatment. As in the primary analyses, using a foreign language attenuated the perceived negativity of adverse effects (*Estimate* = −10.45, *SE* = 2.35, *t*(138.41) = −4.44, *p* < 0.001), which was not moderated by proficiency (*p* = 0.926) or reading ease (*p* = 0.187). Using a foreign language increased sensitivity to population risks when estimating personal risks (*Estimate* = 0.07, *SE* = 0.03, *t*(2780.76) = 2.62, *p* = 0.009), which was not moderated by proficiency (*p* = 0.756) or reading ease (*p* = 0.987). Lastly, using a foreign language increased sensitivity to expected utility when determining willingness to accept preventative treatment (*Estimate* = 0.11, *SE* = 0.04, *t*(1523.4) = 2.50, *p* = 0.012), which was not moderated by proficiency (*p* = 0.177) or reading ease (*p* = 0.733).

## 5. Discussion

The goal of the present experiment was to examine the influence of native and foreign languages on medical judgments. Specifically, we were interested in whether using a native vs. foreign language would alter bilinguals’ subjective evaluations, perceptions of risk, and sensitivity to costs and benefits when making decisions regarding preventative care. Based on prior work demonstrating that information is often processed less emotionally in a non-native language, we hypothesized that potential adverse effects of medical conditions and treatments would be perceived as less negative in a foreign language (Hypothesis 1). This was indeed the case—participants who were randomly assigned to use their foreign language rated both disease symptoms and treatment complications as less negative compared to those using their native tongue. This finding is consistent with research showing that using a foreign language often elicits weaker affective evaluations, including reductions in perceived disgust towards products designed for sustainable consumption [22], diminished feelings of ownership when pricing goods for sale [23], and less extreme affective responses when judging moral transgressions [15]. The present study demonstrates that using a foreign language reduces the perceived severity of health-related outcomes, with potential consequences for how millions of providers and patients make decisions regarding medical care.

Given that greater fear of an outcome can inflate the perceived likelihood that it will occur, e.g., [29,68,69], we further predicted that using a foreign language would attenuate the estimated risk of experiencing adverse effects (Hypothesis 2). Contrary to expectations, however, the estimated probability of negative outcomes was comparable in the native and foreign language. This is in contrast to Hadjichristidis et al.’s [73] finding that potential hazards in non-medical contexts are perceived as less risky when evaluated in a non-native language. This apparent inconsistency could be attributed to at least three key differences between the two studies. First, participants in Hadjichristidis et al.’s experiment were only prompted with the potential hazard (e.g., “nanotechnology”) before making their ratings, while participants in the present study were also told how often a given outcome occurs within the general population. Because access to objective probabilities may reduce reliance on subjective feelings, any further reduction in affective processing due to language may have had a negligible impact on risk perception. Second, while Hadjichristidis et al.’s participants evaluated risk using a language-based scale (i.e., “absolutely not risky”, “not risky”, “slightly risky”, “moderately risky”, “fairly risky”, very risky”, “extremely risky”), participants in the present study were asked to provide numerical estimates (i.e., probabilities from 0 to 100). Compared to providing linguistically coded ratings, generating numerical estimates may engage the linguistic system to a lesser extent and may therefore reduce the effect of native vs. non-native language use on perceived risk. Lastly, and perhaps most critically, participants in Hadjichristidis et al.’s experiment were asked to evaluate the “risk” of potential hazards, while participants in the present study were asked to estimate the “likelihood” of experiencing adverse effects. This distinction is meaningful because the concept of risk is often associated with both the probability and severity of negative outcomes [71]. In other words, while the present study was intentionally designed to elicit separate judgments of likelihood and severity, Hadjichristidis et al.’s measure of risk may reflect a combination of the two constructs. Indeed, the authors found that the effect of language on risk was mediated by ratings of affective responses—using a foreign language elicited less negative and more positive feelings about the potential hazards, which were in turn associated with lower perceived risk and higher perceived benefit. It is therefore possible that the foreign language reduction in perceived “risk” reported by Hadjichristidis et al. primarily stems from a reduction in the perceived severity of negative outcomes rather than a reduction in the perceived likelihood of their occurrence (consistent with the results of the present study).

Despite the minimal effect of language on overall levels of perceived likelihood, we found that participants using a foreign language were more likely to account for the prevalence of an outcome within the general population when estimating their personal likelihood of experiencing the outcome (Hypothesis 3). Research has shown that perceptions of personal risk often do not correspond with the perceived or known risks faced by others [72], which in some cases can result from overoptimism regarding one’s own future [95]. For instance, we may perceive our chances of experiencing a negative event to be less than that of others as a kind of defense mechanism. Using a foreign language could have minimized the need for self-serving optimism, resulting in estimates that were more comparable to the stated population risks (see [96]). And yet, likelihood risk estimates in the native language were not generally lower than population risks, but rather less extreme—objectively low-probability events were perceived as more likely and high-probability events as less likely. Like overoptimism, this type of distorted risk perception may stem from affective responses to potential outcomes [85,86]—fear could make a small probability of a negative event loom larger, while wishful thinking may prompt us to exaggerate a small chance of avoiding it. While it should be noted that participants in both language groups overestimated small probabilities and underestimated large probabilities, the less emotional foreign language may have attenuated the degree of distortion compared to the native language.

Visual inspection of mean risk ratings additionally suggests that the difference between native and foreign language users may be especially pronounced at higher probabilities of adverse effects. Though speculative, such a finding could provide further support for an emotionally mediated account of the foreign language effect, as an affectively-driven effect of language would be expected to be most notable for highly emotional judgments. In the moral domain, endorsement of utilitarian actions (e.g., sacrificing one person to save five) tends to decline with increasing severity of the outcomes (e.g., killing vs. injuring one to save five [97]) and degree of direct contact with the sacrificial victim (e.g., killing one person by pushing them off a bridge vs. flipping a switch to divert the trolley [60]). Both manipulations can affect the emotional aversiveness of the sacrifice, and the foreign language increase in utilitarianism has been shown to be greater for more aversive versions of moral dilemmas [12,13,98] (e.g., for the footbridge [99] than switch [100] version of the trolley dilemma). Similarly, it may be the case that the influence of language on medical judgments will be especially likely to emerge for more emotionally aversive evaluations (e.g., of highly probable disease symptoms and treatment complications).

Finally, we found that using a foreign language increased sensitivity to the combined desirability and likelihood (i.e., “expected utility”) of events when choosing whether or not to accept preventative treatment (Hypothesis 4). Pachur et al. [87] found that more emotional decisions are associated with disproportionate reliance on the relative desirability of possible outcomes without due consideration for how likely they are to occur. Our findings suggest that differences in the emotional resonance of native vs. foreign languages can have an analogous impact on decisions about preventative care. Sensitivity to expected utility, and in particular to the relative risks associated with accepting vs. declining treatment, was enhanced when judgments were made in a non-native tongue.

The influence of language on medical judgment could be driven by a number of potential mechanisms. For instance, increased sensitivity to objective risks and relative costs-and-benefits when using a non-native tongue could stem from either attenuated emotion and automatic “System I” processing or enhanced deliberation and reflective “System II” processing. Hsee and Rottenstreich [85] found that sensitivity to relative quantities of goods when making pricing decisions was greater for individuals who were first asked a series of calculation questions compared to those who were asked about their feelings. Because one group was primed to think more analytically and the other more intuitively, the resulting difference in sensitivity could be attributed to variable reliance on deliberative reasoning, intuition-based feelings, or both.

Though an “enhanced deliberation” account cannot be ruled out in the present experiment, we propose that the observed effects of language on medical judgment are more likely to stem from a reduction in affective processing for two reasons. First, our finding that using a foreign language reduces the perceived negativity of adverse effects is consistent with the hypothesis that information is processed less emotionally in a non-native language. Secondly, despite its theoretical plausibility, there has been relatively little evidence to support the increased deliberation account of foreign language effects. Using a foreign language has not been shown to increase deliberative problem solving on the CRT ([18,101,102]; cf. [13]) and may in fact reduce the capacity to engage in logic-based syllogistic reasoning [55]. Additionally, while using a foreign language has been shown to attenuate emotionally driven biases (e.g., loss aversion [18,20]), similar effects are not observed when judgments are biased by flawed reasoning rather than emotion (e.g., base rate neglect, outcome bias, and representativeness heuristic [102,103]). In the moral domain, studies explicitly designed to disentangle utilitarian considerations (e.g., maximizing the greater good) from supposedly more emotionally grounded “deontological” values (e.g., against actively harming another person) indicate that using a foreign language attenuates the influence of the latter without increasing the former [16,104]. Such findings are largely consistent with the hypothesis that using a foreign language may blunt emotional or intuitive processing without increasing deliberation. Future studies incorporating neural and physiological measures (e.g., fMRI, galvanic skin response, pupillometry, and heart rate) in addition to ratings of perceived negativity and risk could provide converging evidence for the role of emotion in language-dependent medical judgments.

The mechanisms and boundaries of the present findings could also be examined through the use of different bilingual populations and languages. In particular, because language can serve as a powerful prime for culture [105,106,107,108,109], it will be necessary to replicate the results from native-Mandarin, foreign-English speakers with native-English, foreign-Mandarin speakers. For instance, while we attribute the lower negativity ratings in English relative to Mandarin to the use of a foreign language, the possibility remains that such differences emerged from the activation of distinct cultural norms regarding the expression of physical discomfort and pain [110,111]. Though more work is needed to determine the precise mechanisms underlying the observed effects, the present study serves as an initial step to uncover the ways in which using a native vs. non-native language shapes judgment in the medical domain.

Lastly, the results of the present study may serve as a basis for generating new predictions regarding the influence of language on judgment and choice. For instance, a possibility raised by an anonymous Reviewer is that the increased sensitivity to the relative risks of declining vs. accepting treatment in a foreign language may be related to the “sunk cost” phenomenon whereby individuals tend to invest more resources in a course of action if previous investments had already been made [112]. In the moral domain, it has been found that individuals are more likely to endorse aversive actions if a similar decision had already been made [113]. To the extent that the consequences of declining treatment (e.g., disease symptoms) could be considered a prior investment, one may expect that increasing the severity and risk of disease symptoms would increase tolerance of negative consequences associated with the treatment. Though to our knowledge, there is no evidence to suggest that consideration of sunk costs is increased in a foreign language, it may be the case that using a non-native language increases the salience of outcomes associated with different courses of action in the past as well as the present.

## 6. Conclusions

Using a foreign language has been shown to influence how bilinguals think, feel, and behave in domains ranging from moral judgment to financial decision making. The present investigation extends the study of foreign language effects to the medical domain. We find that when using a foreign language, bilinguals perceive medical symptoms and treatment side effects as less severe and are more sensitive to the relative risks of accepting vs. declining preventative treatment. We therefore conclude that use of a native vs. non-native language impacts how we weigh the costs and benefits of preventative treatment when making critical decisions about our personal health. The increasing ubiquity of multilingualism, both among healthcare providers and those who seek treatment, highlights the value and importance of understanding how medical judgments are shaped by language experience and linguistic context. Our findings suggest that language can systematically alter consequential judgments about preventative care, not only by functioning as a vehicle that carries information, but also by shaping how health-related information is processed and perceived.

## Figures and Tables

**Table 1:** Demographics of study participants by language condition.

	Native Mandarin	Non-Native English	*p*
Age	25.93 (4.29)	25.63 (3.55)	0.63
Gender	53% Female	56% Female	0.79
Mandarin AoA	0.77 (1.81)	0.88 (1.87)	0.72
English AoA	6.24 (3.65)	6.00 (3.31)	0.67
Mandarin AoR	3.19 (2.83)	3.07 (2.80)	0.80
English AoR	7.38 (3.64)	7.23 (3.28)	0.78
Mandarin Speaking	9.67 (0.99)	9.49 (1.41)	0.31
English Speaking	7.84 (1.41)	7.26 (1.42)	0.01 *
Mandarin Understanding	9.72 (0.80)	9.63 (0.91)	0.49
English Understanding	8.16 (1.39)	7.86 (1.29)	0.16
Mandarin Reading	9.79 (0.47)	9.61 (0.99)	0.16
English Reading	8.62 (1.08)	8.25 (1.50)	0.08

Numbers represent means and standard deviations (in parentheses). Participants’ age, gender, ages of Mandarin and English acquisition, and self-reported Mandarin and English proficiency in speaking, understanding, and reading were assessed using an abridged version of the *Language Experience and Proficiency Questionnaire (LEAP-Q* [90]). AoA = age of acquisition; AoR = age of reading acquisition. * *p* < 0.05

**Table 2:** Effects of Language on Perceived Negativity and Perceived Risk.

	*Estimate*	*SE*	*df*	*t*	*p*
*Perceived Negativity*					
Language	−10.43	2.22	270.36	−4.70	<0.001 ***
Language:Medical_Event	−3.05	3.28	484.83	−0.93	0.354
Language:Relevance	−3.87	3.28	484.31	−1.18	0.239
Language:Population_Risk	−0.02	0.02	2819.19	−0.93	0.354
Lang:Med:Rel	11.24	8.86	270.53	1.27	0.206
Lang:Med:PopRisk	−0.02	0.04	2824.13	−0.57	0.568
Lang:Rel:PopRisk	0.07	0.04	2824.02	1.52	0.128
Lang:Med:Rel:PopRisk	−0.09	0.09	2819.49	−1.02	0.309
*Perceived Risk*					
Language	−2.14	2.55	266.66	−0.84	0.403
Language:Medical_Event	1.7	3.82	454.25	0.45	0.656
Language:Relevance	−2.74	3.82	453.83	−0.72	0.474
Language:Population_Risk	0.05	0.02	2821.71	2.14	0.032 *
Lang:Med:Rel	11.72	10.22	266.5	1.15	0.252
Lang:Med:PopRisk	0.01	0.05	2823.25	0.26	0.791
Lang:Rel:PopRisk	0.06	0.05	2823.38	1.13	0.257
Lang:Med:Rel:PopRisk	0.09	0.1	2821.55	0.91	0.365

*** *p* < 0.001; * *p* < 0.05

**Table 3:** Effects of Language and Relative Risk/Negativity on Willingness to Accept Preventative Treatment.

	*Estimate*	*SE*	*df*	*t*	*p*
*Relative Negativity*					
Intercept	61.19	3.97	13.02	15.40	<0.001 ***
Language	−2.73	2.99	44.47	−0.91	0.366
Relative Negativity	0.35	0.03	1504.85	11.09	<0.001 ***
Lang:Relative Negativity	0.05	0.04	1277.62	1.16	0.248
*Relative Risk*					
Intercept	62.76	3.97	13.36	15.80	<0.001 ***
Language	−0.59	3.02	46.11	−0.20	0.846
Relative Risk	0.25	0.03	1558.80	9.04	<0.001 ***
Lang:Relative Risk	0.10	0.04	1524.98	2.46	0.014 *

*** *p* < 0.001; * *p* < 0.05.

**Figure A1:**
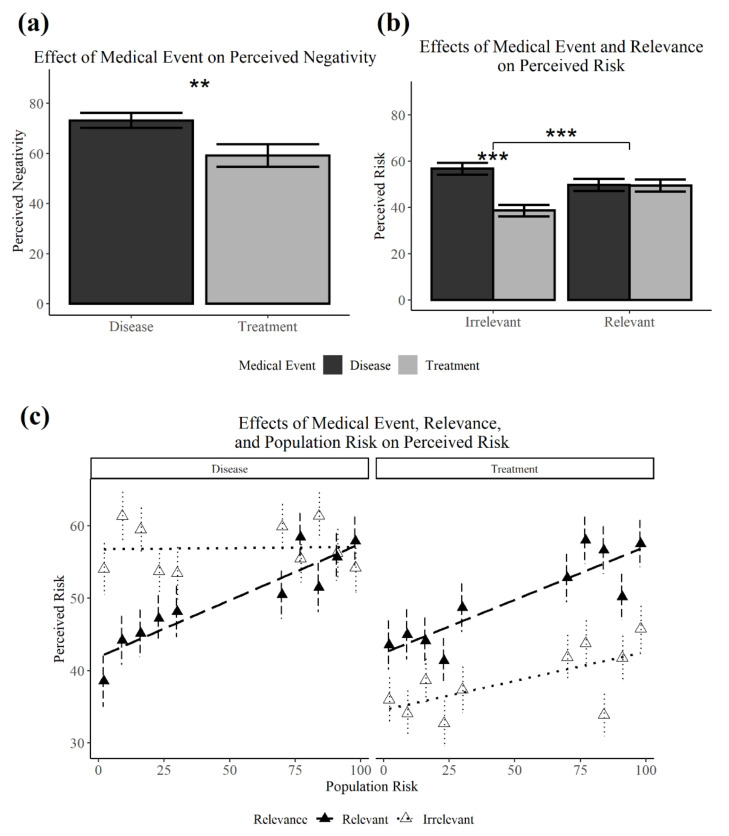
Effects of medical event on (**a**) perceived negativity and (**b**,**c**) perceived risk. The perceived negativity of disease symptoms generally exceeded that of treatment complications (**a**). The perceived risk of disease symptoms exceeded that of treatment complications when participants had knowledge of irrelevant, but not relevant population risks (**b**). The perceived risk of both disease symptoms and treatment complications increased with higher relevant population risks, while only the perceived risk of treatment complications increased with irrelevant population risks (i.e., of the disease; (**c**)). Error bars represent standard error. *** *p* < 0.001, ** *p* < 0.01.

## Data Availability

Data reported in the manuscript are available at https://osf.io/xue6t/, accessed on 21 September 2021.

## Appendix A. Effects of Medical Event on Perceived Negativity, Perceived Risk, and Willingness to Accept Preventative Treatment

**Finding** **A1:**
*Disease symptoms are perceived as more negative than treatment complications.*

Perceived negativity was significantly greater for disease symptoms (*M* = 72.93, *SD* = 14.18) than for treatment complications (*M* = 58.83, *SD* = 16.54; see Figure A1a and Table A1). This effect was not moderated by Language, Population Risk, or Relevance, suggesting that the aversiveness of disease symptoms generally exceeded that of treatment complications.

**Finding** **A2:**
*Disease symptoms are perceived as more likely than treatment complications when provided with irrelevant, but not relevant population risks.*

There was a significant main effect of Medical Event on perceived risk, which was moderated by the relevance of known population risks (see Figure A1b and Table A1). Specifically, the perceived risk of disease symptoms was greater than that of treatment complications when provided with irrelevant (*Estimate* = −18.11, *SE* = 3.33, z = −5.45, *p* < 0.001), but not relevant population risks (*Estimate* = −0.32, *SE* = 3.31, z = −0.10, *p* = 0.924). Lastly, there was a three-way interaction between Medical Event, Relevance, and Population Risk. When provided with relevant population risks, the perceived risk of both disease symptoms (*Estimate* = 0.15, *SE* = 0.03, *t(*744.83) = 6.17, *p* < 0.001) and treatment complications (*Estimate* = 0.13, *SE* = 0.03, *t*(663.18) = 4.97, *p* < 0.001) significantly increased with higher population risks (both *p* < 0.001). When provided with irrelevant population risks, the perceived risk of treatment complications increased with higher disease population risk (*Estimate* = 0.08, *SE* = 0.02, *t*(744.11) = 3.35, *p* < 0.001), while the perceived risk of disease symptoms was unaffected by treatment population risk (*Estimate* = −0.01, *SE* = 0.03, *t*(663.46) = −0.27, *p* = 0.787; see Figure A1c).

**Table A1:** Effects of Medical Event on Perceived Negativity and Perceived Risk.

	*Estimate*	*SE*	*df*	*t*	*p*
*Perceived Negativity*					
Medical_Event	−13.79	3.21	14.13	−4.29	0.001 **
Medical_Event:Language	−3.05	3.28	484.83	−0.93	0.354
Medical_Event:Relevance	6.40	4.42	270.62	1.45	0.149
Medical_Event:Population_Risk	−0.01	0.02	2819.26	−0.33	0.738
Med:Lang:Rel	11.24	8.86	270.53	1.27	0.206
Med:Lang:PopRisk	−0.02	0.04	2824.13	−0.57	0.568
Med:Rel:PopRisk	0.01	0.04	2819.59	0.12	0.906
Med:Lang:Rel:PopRisk	−0.09	0.09	2819.49	−1.02	0.309
*Perceived Risk*					
Medical_Event	−10.48	2.75	23.1	−3.81	0.001 **
Medical_Event:Language	1.7	3.82	454.25	0.45	0.656
Medical_Event:Relevance	23.76	5.1	266.58	4.66	<0.001 ***
Medical_Event:Population_Risk	0.03	0.02	2818.09	1.08	0.28
Med:Lang:Rel	11.72	10.22	266.5	1.15	0.252
Med:Lang:PopRisk	0.01	0.05	2823.25	0.26	0.791
Med:Rel:PopRisk	−0.11	0.05	2821.69	−2.22	0.026 *
Med:Lang:Rel:PopRisk	0.09	0.1	2821.55	0.91	0.365

*** *p* < 0.001; ** *p* < 0.01; * *p* < 0.05.

**Finding** **A3:** *Knowledge of population treatment risks increased willingness to accept preventative treatment relative to knowledge of population disease risks.*.

There was a significant main effect of Risk Condition on willingness to accept preventative treatment (*Estimate* = 5.70, *SE* = 2.62, *t(*99.14) = 2.17, *p* = 0.032). Specifically, individuals who knew the population risk of treatment complications expressed greater willingness to accept preventative care (*M* = 67.82, *SD* = 32.18) compared to those with knowledge of disease risks (*M* = 62.86, *SD* = 34.43). As noted previously, however, Risk Condition did not moderate the effects of Language or Relative Treatment Utility on willingness to accept treatment. Additional follow-up analyses confirm that willingness to accept treatment increased with greater expected harm of the disease (i.e., perceived negativity × perceived risk; *Estimate* = 0.40, *SE* = 0.04, *t(*1566.46) = 10.93, *p* < 0.001) and declined with greater expected harm of the treatment (*Estimate* = −0.39, *SE* = 0.05, *t(*1575.12) = −7.72, *p* < 0.001), neither of which were moderated by Risk Condition (*p* = 0.916 and 0.416, respectively) (see Table A2 and Table A3).

## Appendix B. Full Output for Analyses of Perceived Negativity and Perceived Risk

**Table A2:** Effects of Language, Medical Event, Relevance, and Population Risk on Perceived Negativity.

	*Estimate*	*SE*	*df*	*t*	*p*
(Intercept)	64.48	3.55	10.76	18.17	<0.001 ***
Language	−10.43	2.22	270.36	−4.70	<0.001 ***
Medical_Event	−13.79	3.21	14.13	−4.29	0.001 **
Relevance	−1.95	1.64	438.79	−1.18	0.237
Population_Risk	0.03	0.01	2818.91	2.56	0.010 *
Lang:MedEvent	−3.05	3.28	484.83	−0.93	0.354
Lang:Rel	−3.87	3.28	484.31	−1.18	0.239
MedEvent:Rel	6.40	4.42	270.62	1.45	0.149
Lang:PopRisk	−0.02	0.02	2819.19	−0.93	0.354
MedEvent:PopRisk	−0.01	0.02	2819.26	−0.33	0.738
Rel:PopRisk	0.03	0.02	2821.90	1.37	0.172
Lang:MedEvent:Rel	11.24	8.86	270.53	1.27	0.206
Lang:MedEvent:PopRisk	−0.02	0.04	2824.13	−0.57	0.568
Lang:Rel:PopRisk	0.07	0.04	2824.02	1.52	0.128
MedEvent:Rel:PopRisk	0.01	0.04	2819.59	0.12	0.906
Lang:MedEvent:Rel:PopRisk	−0.09	0.09	2819.49	−1.02	0.309

*** *p* < 0.001; ** *p* < 0.01; * *p* < 0.05.

**Table A3:** Effects of Language, Medical Event, Relevance, and Population Risk on Perceived Risk.

	*Estimate*	*SE*	*df*	*t*	*p*
(Intercept)	44.11	1.87	25.38	23.62	<0.001 ***
Language	−2.14	2.55	266.66	−0.84	0.403
Medical_Event	−10.48	2.75	23.1	−3.81	0.001 **
Relevance	−3.29	1.93	311.8	−1.71	0.089
Population_Risk	0.09	0.01	2819.64	7.23	<0.001 ***
Lang:MedEvent	1.7	3.82	454.25	0.45	0.656
Lang:Rel	−2.74	3.82	453.83	−0.72	0.474
MedEvent:Rel	23.76	5.1	266.58	4.66	<0.001 ***
Lang:PopRisk	0.05	0.02	2821.71	2.14	0.032 *
MedEvent:PopRisk	0.03	0.02	2818.09	1.08	0.28
Rel:PopRisk	0.11	0.02	2817.71	4.25	<0.001 ***
Lang:MedEvent:Rel	11.72	10.22	266.5	1.15	0.252
Lang:MedEvent:PopRisk	0.01	0.05	2823.25	0.26	0.791
Lang:Rel:PopRisk	0.06	0.05	2823.38	1.13	0.257
MedEvent:Rel:PopRisk	−0.11	0.05	2821.69	−2.22	0.026 *
Lang:MedEvent:Rel:PopRisk	0.09	0.1	2821.55	0.91	0.365

*** *p* < 0.001; ** *p* < 0.01; * *p* < 0.05.
